# Identification of two new keratinolytic proteases from a *Bacillus pumilus* strain using protein analysis and gene sequencing

**DOI:** 10.1186/s13568-016-0213-0

**Published:** 2016-06-30

**Authors:** Soltana Fellahi, Abdelwaheb Chibani, Elisabeth Feuk-Lagerstedt, Mohammad J. Taherzadeh

**Affiliations:** Department of Biology, Faculty of Sciences of Nature and Life, Mostaganem University, Mostaganem, Algeria; Swedish Centre for Resource Recovery, University of Borås, Borås, Sweden

**Keywords:** *Bacillus pumilus*, Keratinase, α-Keratin, β-Keratin, NanoHPLC–ESI–MS/MS, DNA sequencing

## Abstract

The *Bacillus* strain (CCUG 66887) has a high capacity to excrete keratinase with the ability to degrade *both* alpha- and beta keratin. In this study we aimed to show the characteristics of the keratinolytic protease and to identify its gene by using liquid chromatography–electrospray ionization tandem mass spectrometry methods (nanoHPLC–ESI–MS/MS) followed by Mascot data base search. The results showed that the enzyme in fact consists of two different keratinases, both with a molecular mass of 38 kDa. Further, DNA sequencing generated the open reading frame (ORF) of one of the genes (*Ker1*), and de novo genome sequencing identified the ORF of the second gene (*Ker2*). The two keratinase genes contain 1153 base pairs each and have a gene similarity of 67 %. In addition, the *Bacillus* strain was classified as *Bacillus pumilus* and its genes were annotated in the GeneBank at NCBI (accession: CP011109.1). Amino acid sequences alignment with known *B. pumilus* proteases indicated that the two keratinases of *B. pumilus* strain C_4_ are subtilisin-like serine proteases belonging to the Protease S8 family. Taken together, these result suggest the two keratinases as promising candidates for enzymatic processing of keratinous wastes in waste refinery.

## Introduction

Annually, just the global feather waste from the poultry processing industry reaches 8.5 million tons. At present, the poultry feathers are dumped, buried, used for land filling, or incinerated, resulting in environmental challenges in terms of storage, handling, emission control, and ash disposal (Agrahari and Wadhwa 2010). Poultry feathers are also turned into feather meal used as animal feed because of the high protein content. However, the use of waste for animal feed is becoming tighter (Commission of the European Communities 2000). Additionally, the high treatment costs make the process economically unfeasible. An environmentally and economically promising process to recover the feather waste is to produce renewable energy by e.g. anaerobic digestion. In this process, not only does the valuable methane result as a byproduct, but also digested residues are formed. The latter can safely be used as a fertilizer, since pathogens presented in the feather waste have been eradicated in the process (Salminen and Rintala 2002a, b).

The recalcitrant keratin is the major compound in several biological materials. It is also the waste product in poultry, slaughterhouse, leather- and fur processing industries and consists of feather, hair, horn, hoof, nails, claws, wool, and bristles (Kornillowicz-Kowalska and Bohacs 2011). While some of the materials like hair or wool, to a great extent, are composed of the helix form of α-keratin, other materials such as feather are largely composed of the flat form of β-keratin. Among the two different types of keratin structures, the content of sulfur varies giving the keratin a softer or harder structure and affects the degradation of the keratinous material to a greater extent (Brandelli et al. 2010). A large number of microorganisms have been reported to produce keratinases (Brandelli et al. 2010; Gupta and Ramnani 2006; Onifade et al. 1998), and among bacteria, the best studied are organisms from the genus *Bacillus* (Gobinath et al. 2014). Keratinases (EC 3.4.99.11) are serine- or metalloproteases (Gupta and Ramnani 2006), and many bacterial keratinases have been sequenced, cloned, and characterized indicating a sequence similarity with the subtilisin family, Family S8, of serine proteases (Rawlings and Barret 1993).

The isolation and characterization of a keratin-degrading bacterium, *Bacillus* sp C_4_, has been reported by this lab. The proteolytic activity was broadly specific, and the bacterium could grow and produced a significant level of keratinase when using wool or chicken feather as substrates. A total hydrolysis of the keratinous waste was obtained in less than 3 days (Fellahi et al. 2014). Also this proteolytic enzyme has shown activity and stability over a broad pH range with two distinct optima, one at pH 8.5 and the other at pH 11, indicating that it might be not one but two enzymes. Its activity was completely inhibited by phenylmethylsulfonyl fluoride (PMSF) pointing out that the enzyme is a serine protease (Fellahi 2009).

In an attempt to increase our understanding of the *Bacillus* strain’s ability to simultaneously hydrolyze *both* α- and β-keratin, we in this study aimed to show the characteristics of the keratinolytic protease and to identify its gene. So far, the vast majority of the identified keratinase-producing organisms appears to be able to hydrolyze only the β-keratin in the chicken feather (Gupta et al. 2013), which gives the keratinolytic protease from this strain a potential for simultaneous degradation of both types of keratin in waste refinery.

## Materials and methods

### Bacterial strain and medium

The microorganism used in this study was *Bacillus* sp. C_4_; CCUG 66887. It has earlier been isolated from the compost and identified using biochemical tests and 16S rDNA technique (GenBank accession: FJ214667) (Fellahi et al. 2014). Before the strain was used for protease production it was grown at 37 °C for 24 h on peptone yeast extract medium containing Bactopeptone, 10 g/l; Yeast extract, 5 g/l and NaCl, 5 g/l.

### Keratinase gene sequence determination

Multiple sequence alignment with CLUSTALW2 (http://www.ebi.ac.uk/Tools/msa/clustalw2/) (Chenna et al. 2003) was used to align keratinase genes from different *Bacillus pumilus* strains to investigate the resemblance among the strains to be able to choose one strain for designing the first sequencing primer set (F: TTAGAAGCCGCTTGAACGTTA, R: ATGTGCGTGAAAAAGAAAAATGTG). Genomic DNA was isolated from strain C_4_ using MasterPure™ Gram Positive DNA Purification Kit (Epicentre), and the DNA was sent together with the sequence for the first primer set to Eurofins Genomics, Germany where the primers were synthesized and both DNA strands sequenced by Sanger method. From the retrieved two DNA sequences, a new primer set (F: AAGTATTAGATCGTTACGGCGATGGAC, R: CCAAGAACACCAATCGTGTTATCAAGG) was designed and once again sent to Eurofins Genomics together with genomic DNA. This procedure was repeated a third time with primer (F: TTGCCAACGTGAACAGCAAC) to determine the open reading frame (ORF) of the gene.

### De novo sequencing and genome annotation

To be able to search the genome of strain C_4_ for additional putative keratinase genes, de novo sequencing of the whole genome using the instrument MiSeq and the MiSeq Control Software 2.3.0.3 was performed by Eurofins Genomics, Germany. The sequence assembly and scaffolding was done using the Newbler assembler software v2.9. The genome sequence was annotated using the prokaryotic annotation pipeline at the National Center for Biotechnology Information, Bethesda, USA (NCBI).

### Partial purification of proteases for nanoHPLC–ESI–MS/MS

The protease production from *Bacillus* C_4_ strain was done according to Fellahi and coworkers. (Fellahi et al. 2014). In short: the C_4_ strain was grown in 50 ml of modified Schaeffer’s medium (Leighton and Doi 1971) containing Beef extract, 3 g/l; Bactopeptone, 5 g/l; KCl, 2 g/l; Yeast extract, 2 g/l; pH 7, and supplemented with 2 mM MgSO_4_.7H_2_O; 1 mM CaCl_2_; 0.1 mM MnCl2; 1 mM FeSO_4_; and 0.1 % (w/v) glucose. The production was done using a 250-ml E-flask with a 2 % inoculum size in a shaker incubator (Excella 24, New Brunswick Scientific) at 37 °C, 160 rpm. After 24 h, the cell-free supernatant was received by centrifugation at 8000 rpm for 15 min at 4 °C (Optima Max-XP, Beckman Coulter).

For the identification of the enzyme by nanoHPLC–ESI–MS/MS the cell-free supernatant was precipitated by adding NH_3_SO_4_ to 65 % saturation at 4 °C and slowly mixing in a shaker incubator (Excella 24, New Brunswick Scientific) for 1 h. The precipitate was collected by centrifugation at 12,000 rpm for 30 min at 4 °C (Optima Max-XP, Beckman Coulter). The pellet was re-suspended in 500 µl of 20 mM Tris–HCl buffer, pH 8, followed by dialysis overnight against the same buffer. Proteolytic activity was measured as described by Cliffe and Law (1982), using Hide Powder Azure (HPA, Sigma) as substrate. Approximately 20 µg of the purified enzyme was run on a 12 % SDS-PAGE-gel, according to Laemmli (1970) along with a molecular weight protein marker (All Blue Protein Precision Standard, Bio-Rad).

### Protein identification by nanoHPLC–ESI–MS/MS and data base search

Protein identification of the crude enzyme was performed by Proteome Factory AG, Germany. Two protein spots from a 12 % SDS-PAGE-gel were cut out and digested in-gel by trypsin (Promega, Mannheim, Germany) and analyzed by nanoHPLC–ESI–MS/MS. The LCMS system consisted of an Agilent 1100 nanoHPLC system (Agilent, Waldbronn, Germany), PicoTip electrospray emitter (New Objective, Woburn, MA, USA), and an Orbitrap XL or LTQFT. The retrieved peptides were analyzed using an ultra-mass spectrometer (ThermoFisher Scientific, Bremen, Germany). Peptides were first trapped and desalted on the enrichment column Zorbax 300SB-C18, 0.3 mm × 5 mm (Agilent) for 5 min (solvent: 2.5 % acetonitrile/0.5 % formic acid), then separated on a Zorbax 300SB-C18, 75 μm × 150 mm column (Agilent) using a linear gradient from 10 to 32 % B (Solvent A: 5 % acetonitrile in water, Solvent B: acetonitrile. Both solvents contained 0.1 % formic acid). Ions of interest were data-dependently subjected to MS/MS according to the expected charge state distribution of the peptide ions. MS/MS ion search of the Mascot search engine (Matrix Science, London, England) was performed, and only peptide matches with a score of 20 or above were accepted. Proteins were identified against the *B. pumilus* entries from the RefSeq protein database available at NCBI, which was appended to an existing bacterial database. The search results were also run against the amino acid sequences retrieved from the *Ker1* and *Ker2* genes.

### Amino acid sequence alignment

The amino acid sequences of the retrieved enzymes from the ultra-mass spectrometer analysis were compared to other proteases produced by the *B. pumilus* strains using the Basic Local Alignment Search Tool Blastp 2.2.3.1 available at NCBI.

## Results

### Keratinase gene sequence determination

The multiple sequence alignment followed by synthesis of primers and Sanger sequencing resulted in an ORF of a keratinase gene, named *Ker1*, which contains 1153 base pairs. The gene was subsequently submitted to the NCBI GenBank (Accession Number: KX184831).

### De novo sequencing and genome annotation

The de novo sequencing of the whole genome indicated that the organism is a *B. pumilus* and that it possesses a genome of 3.6 million base pairs. The organism was annotated to NCBI (Accession Number: CP011109.1) using the prokaryotic annotation pipeline, and the result indicated that this *B. pumilus* strain has around 4000 genes. One of these genes is a homolog to *Ker1* and was named *Ker2.* The gene was subsequently submitted to NCBI GenBank (Accession Number: KX184832).

### Partial purification of proteases from strain C_4_ for nano-HPLC-ESI-MS/MS

**Fig. 1:**
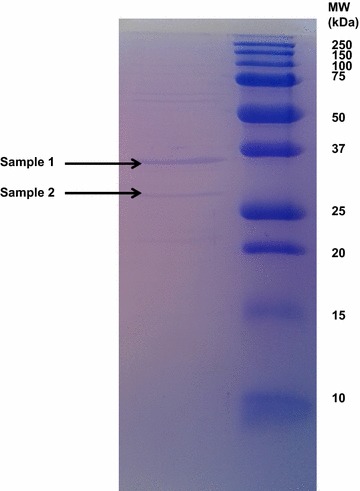
The dialyzed proteolytic enzyme investigated on SDS-PAGE using a 12 % gel. Two bands at 36 and 28 kDa, respectively, were excised for protein identification

The crude protease fraction showed after SDS-PAGE and staining with Coomassie brilliant blue R-250 one distinct band and one weaker band with molecular weights of about 28 and 36 kDa, respectively (Fig. 1).

### Protein identification by nanoHPLC–ESI–MS/MS and data base search

The Mascot Search Result using the *B. pumilus* entries from the RefSeq protein data base and the amino sequences retrieved from the *Ker1* and *Ker2* genes can be seen in Table 1. The band corresponding to 28 kDa only contained keratinase 1, while the band corresponding to 38 kDa contained keratinase 1 and keratinase 2. These results indicated that the partial purified keratinolytic protease in fact consists of two enzymes, both with a molecular weight of 38 kDa, and corresponding to the identified genes *Ker1* and *Ker2* of the *B. pumilus* strain C_4_. When using the *B. pumilus* entries from the RefSeq protein data the peptides from MS/MS identified Peptidase S8. The result also showed a resemblance between keratinase 2 and peptidase S8.

**Table 1:** Protein identification by MASCOT Search on the purified enzyme from *Bacillus* strain C_4_ using RefSeq protein data base and the two amino acid sequences retrieved from *Ker1* and *Ker2*

SDS-Page lane	Identified protein	Sequence identity	MW (kDa)	MASCOT peptides identified	MASCOT ion score^a^
1 and 2	Keratinase 1	WP_008348814.1	38.8	QRLENTATPLGNSFYYGK	81
				GVVVVAAAGNSGSTGSTSTVGYPAK	146
				YDSTIAVANVNSNNVR	122
				LENTATPLGNSFYYGK	102
				GLINVQAASN	85
1	Keratinase 2	WP_017357922.1	38.3	VGVVGVAPK	61
				VADENGDGYYSWIIK	80
				SGTSMASPHVAGAAAVILSK	107
				HPNLTNDELR	53
				HPNLTNDELRER	34
1	Peptidase S8	gi|648268958	38.8	VGVVGVAPK	61
				VADENGDGYYSWIIK	80
				LGEPFYYGAGLVNVQK	106
				SGTSMASPHVAGAAAVILSK	107

^a^Individual ion scores >26 indicates identity or extensive homology at 95 % level of confidence

**Fig. 2:**
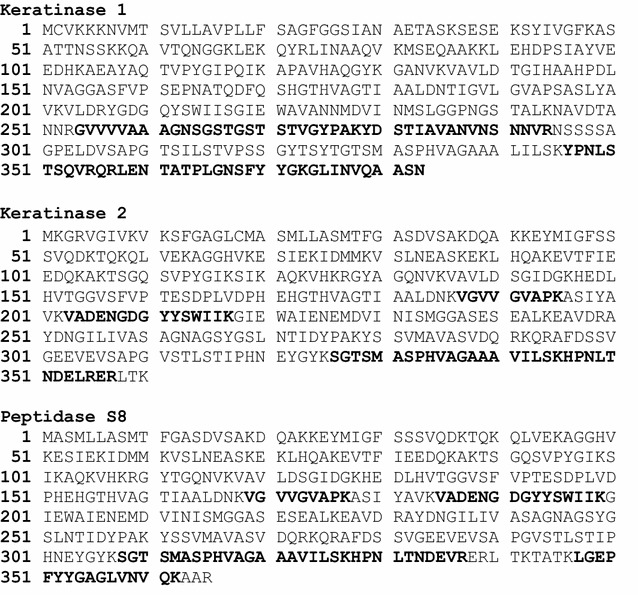
The distribution of peptides from MS/MS in keratinase 1, keratinase 2 and peptidase S8. Matched peptides are shown in *bold black*

Figure 2 illustrates the peptides from Ms/Ms identified in keratinase 1, keratinase 2 as well as peptidase S8 and their distribution and coverage in the respective enzymes. As can be seen keratinase 2 shares three peptides with peptidase S8 but also has unique one while keratinase 1 does not have any peptides in common with the identified Peptidase S8.

### Amino acid sequence alignment

By comparing the amino acid sequences of the two retrieved keratinase enzymes with known proteases of other *B. pumilus* strains the results indicated that these enzymes are subtilisin-like serine proteases belonging to the Protease S8 family (Tables 2, 3).

**Table 2:** Amino acid sequence alignment of keratinase 1 with proteases of *Bacillus* strains using Blastp 2.2.3.1

Description	Organism	Amino acid sequence identity (%)	Accession number
Alkaline serine proteinase	*B. pumilus*	100	ACO94164.1
Lehensis serine protease	*B. lehensis*	100	AFP23380.1
Alkaline serine protease	*B. pumilus*	99	BAE79641.1
Dehairing protease precursor	*B. pumilus*	99	AAR19220.1
Peptidase S8	*B. pumilus*	99	WP_026050071.1
Alkaline serin proteinase	*B. pumilus*	99	BAA93474.1
Protease	*B. pumilus*	99	ADK63096.1
Peptidase S8	MULTISPECIES: *Bacillus*	99	WP_008348814.1
Serine alkaline protease (subtilisin)	*B. stratosphericus LAMA585*	99	EMI14709.1
Subtilisin Carlsberg	*Bacillus pumilus* ATCC 7061	99	EDW22774.1
Peptidase S8	*B. pumilus*	99	WP_034620013.1
Peptidase S8	*B. pumilus*	99	WP_041093123.1
Peptidase S8	*B. stratosphericus*	99	WP_039962807.1
Peptidase S8	*B. invictae*	99	WP_045034875.1
Peptidase S8	*B. pumilus*	99	WP_044140726.1
Peptidase S8	*B. aerophilus*	99	WP_041507592.1
Peptidase S8	*B. altudinis*	99	WP_039167642.1
Peptidase S8	*B. pumilus*	98	WP_012009474.1
Serine alkaline protease	*B. circulans*	98	ADN04910.1
Serine alkaline protease (subtilisin E)	*B. pumilus*	98	KIL22204.1
Serine alkaline protease, preproprotein	*B. pumilus*	98	CAO03040.1
Peptidase S8	*B. * sp. DW5-4	98	WP_034323660.1
Peptidase S8	*B. pumilus*	98	WP_041117216.1
Peptidase S8	*B. safensis*	98	WP_034282323.1
Peptidase S8	*B. pumilus*	98	WP_034663897.1
Serine alkaline protease (subtilisin E)	*B. pumilus*	97	KIL10386.1
Peptidase S8	*B. pumilus*	97	WP_041110188.1
Peptidase S8	*B. * sp. WP8	96	WP_039183048.1
Keratinase precursor	*B. pumilus*	96	ACM47735.1
Serine alkaline protease (subtilisin E)	*Bacillus* sp. HYC-10	96	KIL09959.1
Peptidase S8	*B. xiamenensis*	96	WP_008359041.1
Organic solvent tolerant protease	*B. pumilus*	96	AAU88064.1
Keratinase	*B. pumilus*	96	ADK11996.1
Peptidase S8	*B. safensis*	96	WP_029706931.1
Peptidase S8	*B. pumilus*	96	WP_041089929.1
Peptidase S8	*B. safensis*	96	WP_044335827.1
MULTISPECIES: peptidase S8	*Bacillus*	96	WP_025093353.1
Peptidase S8	*B. pumilus*	95	WP_024426548.1
Serine alkaline keratinase	*Brevibacillus brevis*	95	AGO58466.1
Alkaline serine protease precursor	*B. pumilus*	95	ACM07731.1
Serine alkaline keratinase	*B. circulans*	94	AGN91700.1

**Table 3:** Amino acid sequence alignment of keratinase 2 with proteases of *Bacillus* strains using Blastp 2.2.3.1

Description	Organism	Amino acid sequence identity (%)	Accession number
Serine alkaline protease (subtilisin E)	*B. stratosphericus* LAMA 585	100	EMI12150.1
Peptidase S8	MULTISPECIES: *Bacillus*	100	WP_035390997.1
Subtilisin Carlsberg	*B. sp.* M 2-6	99	EIL84986.1
Peptidase S8	MULTISPECIES: *Bacillus*	99	WP_034647494.1
Peptidase S8	*B. pumilus*	99	WP_029575389.1
Subtilisin Carlsberg	*B. pumilus*	99	KIL26870.1
Peptidase S8	*B. altitudinis*	99	WP_035702958.1
Peptidase S8	*B. pumilus*	99	WP_026050107.1
Pubtilisin	*B. altitudinis* 41KF2b	99	KDE30915.1
Peptidase S8	*B. sp.* DW5-4	95	WP_034319985.1
Peptidase S8	*B. pumilus*	95	WP_044141213.1
Peptidase S8	*B. pumilus*	94	WP_034620505.1
Peptidase S8	*B. pumilus*	94	WP_034661158.1
Subtilisin Carlsberg	*B. pumilus*	94	KIL17080.1
Subtilisin Carlsberg	*B. pumilus* ATCC7061	94	EDW21217.1
Peptidase S8	*B. pumilus*	93	KDE52880.1
Peptidase S8	*B. safensis*	93	WP_034622393.1
Peptidase S8	MULTISPECIES: *Bacillus*	93	WP_029708034.1
Peptidase S8	*B. pumilus*	93	WP_041117873.1
Peptidase S8	*B. safensis*	93	KEP30825.1
Peptidase S8	*B. safensis*	93	WP_029706051.1
Peptidase S8	*B. pumilus*	93	WP_041109684.1
Pubtilisin Carlsberg	*B. pumilus*	93	KIL21504.1
Pubtilisin Carlsberg	*B. pumilus*	92	KIL11523.1
Peptidase S8	*B. sp.* HYC-10	92	WP_008361817.1
Peptidase S8	*B. safensis*	92	WP_034280781.1
Peptidase S8	*B. sp.* WP8	92	WP_039183179.1
Subtilisin	*B. safensis* FO-36b	92	KDE29455.1
Peptidase S8	*B. safensis*	91	WP_046312283.1
Peptidase S8	*B.pumilus*	91	WP_041086842.1
Subtilisin Carlsberg	*B. pumilus*	90	KIL15277.1

## Discussion

The fast growth rate of the microorganisms, the accessibility for genetic engineering and the short time for the production and purification steps make them the ideal source for production of proteases (Rao et al. 1998). The far most popular source of commercial alkaline proteases is from the *Bacillus* species. The main reason for this is their ability to produce large amounts of alkaline proteases having significant proteolytic activity and stability at high pH as well as high temperature (Jacobs 1995; Yang et al. 2000). So far, the vast majority of the identified keratinase producing organisms appear to be able to hydrolyze only β-keratin in chicken feather and few are known to hydrolyze both α- and β-keratin. (Gupta et al. 2013).

In this study two keratinases from the *Bacillus* sp C_4_ strain were identified. The project started by trying to find a keratinase gene in the genome of the strain by attaching primers designed for the *Ker A* gene of *Bacillus licheniformis* PWD-1 identified by Lin and coworkers (1995). After conventional PCR followed by agarose gel electrophoresis we found many different gene products due to unspecific binding of the primers but no gene product comparable in size with a keratinase gen. Still we decided to DNA sequence three fragments by the Sanger method which resulted in three DNA sequences with the lengths of 236–396 base pairs. By using NCBI blast network service (http://www.blast.ncbi.nlm.nih.gov/Blast.cgi), we found that these gene sequences might belong to the genome of a *B. pumilus* strain. With this information at hand we aligned genes from twelve different *B. pumilus* strains (Table 4) using CLUSTAL W2 (http://www.ebi.ac.uk/Tools/msa/clustalw2/). The outcome of the process indicated high resemblance between many sequences, mostly in the beginning and the end of the different aligned genes. One of the strains, *B. pumilus* strain A1, produces a keratinase (Fakhfakh-Zouari et al. 2010) which has comparable qualities with the keratinolytic enzyme produced by *Bacillus* sp C_4_ (Fellahi 2009). Both enzymes are active and stabile over a broad pH range and also their activity is completely inhibited by PMSF suggesting that they both are serine proteases. The keratinase precursor gene from the *B. pumilus* strain A1 was for this reason chosen for primer design of the first pair of sequencing primers for the keratinase gene. After Sanger sequencing the complete ORF of the keratinase gene (*Ker1*) was identified. To find resemblance with other genes from *B. pumilus* strains, nucleotide BLAST was used. The gene showed a high similarity to several serine protease genes from different *B. pumilus* strains. As much as 99 % similarity was found with the peptidase S8 gene of the *B. pumilus* strain W3, GenBank accession: CP011150.1 (Zheng-Bing et al. 2015). This result confirmed that the isolated fragment of the genome encodes one of the serine protease from the *Bacillus* sp. C_4_ strain. By de novo sequencing and annotation of the genome, we could identify a homolog to the peptidase S8 gene, the *Ker2* gene with equal amount of base pairs as *Ker1.* The two genes had a gene sequence similarity of 67 %.

**Table 4:** *Bacillus pumilus* strains used for gene alignment in CLUSTAL W2

*B. pumilus s*train	GI number and accession version	Gene
NJM4	gi|226938414|gb|FJ869878.1|	Alkaline serine proteinase
N/A	gi|38373993|gb|AY458140.1|	Dehairing protease precursor
N/A	gi|7415641|dbj|AB029082.1|	Alkaline serine proteinase
SG2	gi|301131525|gb|GQ398415.1|	Protease gene
SGMM8	gi|290472378|gb|GU143024.1|	Protease (Alp) gene
bppA	gi|87886606|dbj|AB211527.1|	Alkaline serine
A1	gi|222353759|gb|FJ619651.1|	Keratinase precursor
115b	gi|52843271|gb|AY743586.1	Organic solvent protease gene
KS12	gi|300429855|gb|HM219183.1|	Keratinase gene
TMS55	gi|221193393|gb|FJ584420.1|	Alkaline serine protease precursor
3–19	gi|297342830|gb|AY754946.2|	Subtilisin like serine
sapB	gi|186928863|emb|AM748727.1|	Serine alkaline protease preprotein

The crude protease showed after partial purification followed by SDS-PAGE-gel and staining with Coomassie brilliant blue R-250 one distinct band and one weaker band. Their molecular weights were estimated to about 28 and 36 kDa, respectively, when comparing with the molecular weight marker. When the bands on the gel were analyzed by a nanoHPLC–ESI–MS/MS system the result indicated two proteins with a molecular weight of 38. 8 and 38.3 kDa, respectively (Table 1). The molecular size difference between the excised gel bands and the molecular size of the identified enzymes may be due to a degradation prior to the SDS-PAGE. When the proteins were identified by Mascot Search the result indicated that the two proteins in fact are corresponding to the two genes *Ker1* and *Ker2.* That the crude enzyme in fact contains two different keratinases is in agreement with earlier conclusions (Yamamura et al. 2002). Yamamura and his group found that for the bacterium *Stenotrophomonas* sp D-1 it was not sufficient with one protease for effective keratin degradation but two different keratinases were needed. This suggests that degradation requires the cooperative action of multiple enzymes. We are well aware that *Bacillus* sp C_4_ may need more than two keratinases for optimal degradation although we have not found any additional.

When comparing the amino acid sequences of the two retrieved keratinases with known proteases of other *B. pumilus* strains using Blastp we found that keratinase 1 and 2 are subtilisin-like serine proteases belonging to the Protease S8 family (Tables 2, 3). These proteases show broad substrate specificity, have usually a molecular mass in the range 18–90 kDa. They are generally active at neutral and alkaline pH, with optima at pH 7–11 (Rao et al. 1998) and irreversibly inhibited by PMSF (Powers et al. 2002). This is in agreement with our earlier findings (Fellahi 2009).

As a conclusion, the present study confirmed that the keratinolytic protease produced by the non-genetically modified *B. pumilus* strain C_4_ consists of two different enzymes belonging to the Protease S8 family. This may explain why the strain is able to simultaneously hydrolyze both α- and β-keratin in less than three days. It also makes the bacterium a potent candidate in a cost effective pretreatment step of keratinase rich waste in waste refinery as the two waste fractions, avian feather and sheep wool, do not have to be separated before hydrolyzation of the protein into valuable feedstuff for the biogas production.
